# Regular napping mitigates the onset of cognitive fatigue and protects against stress–inflammatory responses to real-world sleep debt

**DOI:** 10.1016/j.bbih.2026.101390

**Published:** 2026-10-13

**Authors:** Sarah Belhout, Laura Poudevigne, Lili Mouton, Aurélie Servonnet, Christine Le Coz, Mariette Gaudin, Mounir Chennaoui, Damien Léger, Arnaud Rabat, Brice Faraut

**Affiliations:** a Université Paris Cité, VIFASOM (UMR Vigilance Fatigue Sommeil et Santé publique), Paris, France; b Institut de Recherche Biomédicale des Armées, Brétigny-Sur-Orge, France; c APHP, Hôpital Hôtel-Dieu, Centre du Sommeil et de la Vigilance, Paris, France

**Keywords:** Naturalistic sleep debt, Napping, Cognitive fatigue, Sustained attention, Time on task effects, Stress and inflammatory biomarkers, BDNF

## Abstract

Chronic sleep debt impairs cognitive processes, stress regulation, and immune homeostasis, while sustained cognitive engagement induces mental fatigue. However, it remains unclear how chronically insufficient sleep in real-life conditions accelerates the onset of mental fatigue during prolonged cognitive activity—and whether daytime napping can buffer these effects. This study investigated the combined effects of chronic sleep debt and extended cognitive engagement on cognitive fatigue and salivary neuro-immune and neurotrophic markers in an ecologically valid context.

Compared with controls, participants with chronic sleep debt showed pronounced declines in Psychomotor Vigilance Task performance (reaction time and number of lapses) across both *time-on-task* and *time-on-week and* showed higher commission rates in the executive Go-NoGo task. These morning impairments were partially attenuated in the participants who practiced afternoon chronic naps, and the reduction in lapses was correlated with nap frequency. Biologically, chronic sleep debt was associated with a blunted morning cortisol response, an increase in IL-1β and IL-6, and a reduction in BDNF levels, but not observed in either the *Control* or *Sleep-Debt + Nap* groups. In the *Sleep-Debt* group, IL-1β levels were positively correlated with sustained attention deficits.

Chronic sleep debt increased attentional fatigability without exacerbating executive decline, an effect that may be driven by neuro-inflammatory processes and effectively alleviated by regular daytime napping. Importantly, these findings indicate that chronic afternoon napping may confer protective and long-lasting restorative benefits observable in morning assessments, reflecting effects that persist over a 24-h period.

## 1 Introduction

In daily life, individuals are continually required to make decisions of varying complexity and urgency within social and professional contexts—tasks that rely heavily on the integrity of attentional and executive functions (Diamond, 2013). Mental fatigue manifests behaviorally through increased error rates, slower reaction times, and reduced adaptive responses (Van der Linden and Eling, 2006; Lorist et al., 2005; Langner et al., 2010; Faber et al., 2012). Traditionally investigated in prolonged high-demand cognitive tasks (Pessiglione et al., 2025; Lim et al., 2010; Möckel et al., 2015 ; Gui et al., 2015), mental fatigue has nonetheless rarely been studied in conjunction with naturally accumulated sleep pressure—even though chronic sleep debt may amplify mental fatigue (Rabat et al., 2016; Ellenbogen, 2016; Belenky et al., 2003).

Whereas sleepiness reflects an increased homeostatic drive for sleep and a diminished capacity to sustain wakefulness (Cajochen and Schmidt, 2025), mental fatigue is primarily characterized by a subjective sense of cognitive exhaustion, reduced mental energy and performance efficiency (Pessiglione et al., 2025; Balkin et al., 2023). What are the physiological consequences of chronic sleep debt and insufficient recovery time? Historically, research on the effects of chronic sleep restriction has primarily focused on sleepiness, attention, memory impairments, and accidental risk (Van Dongen et al., 2003; Dinges et al., 1997; Belenky et al., 2003; Cohen et al., 2010; Rabat et al., 2016; Bougard et al., 2018). While these domains remain critical, accumulating evidence suggests that chronic sleep restriction also disrupts neuroendocrine and immune system homeostasis, with important clinical and public health implications (Faraut et al., 2012). Data from the 2017 Santé Publique France Barometer show that 17% of adults exhibit moderate sleep debt (weekday–weekend sleep difference of 60–120 min), while severe sleep debt (>120 min) is especially prevalent among young adults aged 18–24 years (25–30%) (Léger et al., 2020). This common week-end recovery strategy—extending sleep duration on non-working days—appears insufficient to fully compensate for the negative physiological effects of weekday sleep restriction (Depner et al., 1997). Consequently, it is crucial to differentiate between the physiological state following weekend's “recovery” and the state of cumulative fatigue reached by the end of a working week. Furthermore, napping during the week remains relatively uncommon in this population: only 24.3% of individuals aged 18–24 and 22.9% of those aged 25–34 reported regular weekday napping, with an average duration of approximately 1 h (Léger et al., 2020). Yet, given its potential restorative benefits, napping may represent an underutilized strategy to mitigate the impact of chronic sleep loss among young adults.

Accumulating evidence suggests that chronic sleep debt should be conceptualized as a chronic, sterile inflammatory stressor (McEwen, 2006). This persistent state of sleep restriction disrupts the delicate neuro-endocrine and neuro-immune crosstalk, notably by dysregulating the hypothalamic-pituitary-adrenal (HPA) axis, reflected in a blunted morning cortisol response (Charmandari et al., 2005; Faraut et al., 2020), and triggering a systemic low-grade inflammatory response (Faraut et al., 2012). Pro-inflammatory cytokines, particularly Interleukin-1β (IL-1β) and Interleukin-6 (IL-6), are constitutively expressed in the central nervous system where they function as potent neuromodulators of sleep-wake regulation and cognitive function (Irwin, 2019; Imeri and Opp, 2009; Krueger et al., 2001). When chronically elevated, these inflammatory signals can induce behavioral changes resembling “sickness behavior”, characterized by psychomotor slowing and cognitive impairment (Dantzer, 2018; Martins and Harrison, 2025). When sustained over time—as observed in chronic inflammation—neurobiological alterations driven by inflammatory processes in the brain may contribute to psychopathological manifestations, including depressive symptoms and psychomotor deficits (Dantzer, 2018; Singh et al., 2025; Martins and Harrison, 2025). Moreover, such an inflammatory environment is hypothesized to compromise synaptic plasticity by suppressing neurotrophic support, thereby contributing to the “biocognitive fatigue”, a state where sustained mental effort intersects with a progressive depletion of neuro-biological resources (Singh et al., 2025).

To date, no study has objectively examined the onset and progression of cognitive fatigue arising from the interaction of two key factors: a prolonged cognitive demand, characterized by sustained engagement (analyzed through the time on task effect across 30 min) of multiple cognitive domains, including sustained and selective attention as well as executive functions, and a chronic sleep debt, defined here as a naturally accumulated sleep restriction over an extended period of several weeks. The objective of the present study is to adopt an integrative physiological framework that considers both cognitive functioning and stress–immune system interactions. Unlike tightly controlled laboratory studies, our research was conducted in ecologically valid settings, leveraging continuous online sleep monitoring over a seven-week period to assess the generalizability of experimental sleep deprivation findings to naturalistic contexts. This study aims to elucidate the associations between habitual sleep patterns, stress system functioning, and immune balance under sustained cognitive demand. Our sample consists of individuals from the general population identified as experiencing chronic sleep debt in their everyday lives.

The objectives of the present study are threefold: (i) to characterize the cognitive processes most vulnerable to the combined effects of prolonged mental exertion and chronic sleep debt, and to determine whether cumulative sleep debt amplified the Time-on-Task effect (ToT), resulting in a greater decline in task performance, particularly when sleep debt reaches its peak (i.e., on Friday); (ii) to investigate the neurobiological correlates of this interaction, focusing on salivary biomarkers of stress and immune-related signaling (cortisol, IL-1β and IL-6), to identify potential mechanisms underlying cognitive fatigability; and (iii) to assess the potential restorative benefits of habitual afternoon napping on next-morning assessments, reflecting effects that persist over a 24-h period, hypothesizing that napping limits the performance degradation typically observed through a working week.

## 2 Materials and methods

### 2.1 Participants and ethics approval

Thirty-two healthy volunteers (23F, 9M; 26 right-handed; aged 27.9 ± 6.76 years; mean ± SEM), with an upper and lower bound of 18 and 40 years old), with a normalized Body Mass Index (22.7 ± 3.4 kg/m^2^) participated in these seven weeks combined at home and laboratory-controlled protocol, which was conducted according to the principles laid out in the Declaration of Helsinki of 1975, as revised in 2001. The ethics committee approved the protocol (N°ID RCB: n°2022-A00698-35). The Drug safety national agency (ANSM) was informed of this decision approval. After providing written informed consent, participants underwent a brief medical history with physical examination and completed a series of relevant questionnaires *(see Supplementary materials)*. Financial compensation was provided to all participants.

### 2.2 Experimental design

In this study, 75 healthy volunteers with comparable academic backgrounds were recruited after receiving detailed information about the study protocol and potential risks. Participants provided written informed consent for long-term real-life observation. Each participant was equipped with an actiwatch (AW; Cambridge Neurotechnology Ltd.) to assess sleep–wake behaviors. Participants were also required to complete a daily sleep diary throughout the study. Participants were selected on two sleep profiles: (i) a “Sleep-Debt” profile, defined by a total sleep time on weekdays (Sunday night to Thursday night) at least 90 min shorter than their total sleep time on weekend nights (Friday and Saturday nights); or (ii) a “Control” profile, where total sleep time during weekdays and weekend differed by no more than 45 min, with a minimum of 7 h of sleep on weekdays and weekend. Following the two-week habituation period (P1), 37 of the 75 initially enrolled participants met the predefined inclusion criteria. Participants were excluded for several reasons. These included a total sleep time (TST) of less than 7 h among participants assigned to the Control profile, the absence of the expected increase in TST on weekends relative to weekdays among participants assigned to the Sleep-Debt and Sleep-Debt + Nap profiles, as well as medication use, withdrawal from the study, or scheduling incompatibilities. Additional data-quality and eligibility considerations further reduced the final sample to 32 participants included in the analyses, stratified into three groups: Control (n = 10; 6 women; 27.2 ± 5.9 years), Sleep-Debt (n = 13; 9 women; 27.7 ± 6.3 years), and Sleep-Debt + Nap (n = 9; 8 women; 28.8 ± 8.7 years). The Sleep-Debt + Nap group comprised Sleep-Debt participants who habitually napped around the early-to-mid afternoon and were encouraged, without any imposed requirement, to maximize their napping opportunities during the experimental weekdays as a means of counteracting their sleep debt (up to 60 min of napping time in bed). Consistent with the ecological design of this study, no experimental sleep restriction or external sleep-wake constraints were imposed. During the second phase (P2, 5 weeks; Fig. 1), daily sleep diaries and actigraphy monitoring continued until the end of Week 7 (Fig. 1), ensuring combined continuous ecological and laboratory data collection. Assessments were conducted on Mondays to capture recovery following the weekend and on Fridays to evaluate cumulative sleep debt accrued over the week as well as potential compensatory effects of napping. Laboratory sessions were conducted during Week 3 and Week 7. Each session began at 9:00 a.m. with a saliva sample. Because the duration of wakefulness prior to testing may independently influence fatigability, we examined wake times on testing days across the three experimental groups. Wake times were comparable between groups (Control: 7h12 ± 0h31; Sleep-Debt: 7h11 ± 0h35; Sleep-Debt + Nap: 7h24 ± 0h38; mean ± SEM), with no significant between-group difference (Kruskal–Wallis test, *p* = 0.238). Thus, differences in the duration of wakefulness prior to testing are unlikely to account for the between-group differences observed in fatigability measures. Subsequently, participants completed two 30-min computerized cognitive tasks: the Psychomotor Vigilance Task and the Go-NoGo task, designed to replicate sustained daily cognitive demands. In addition, standardized self-report scales were also completed before and after each task to assess subjective alertness and fatigue.

**Fig. 1:**
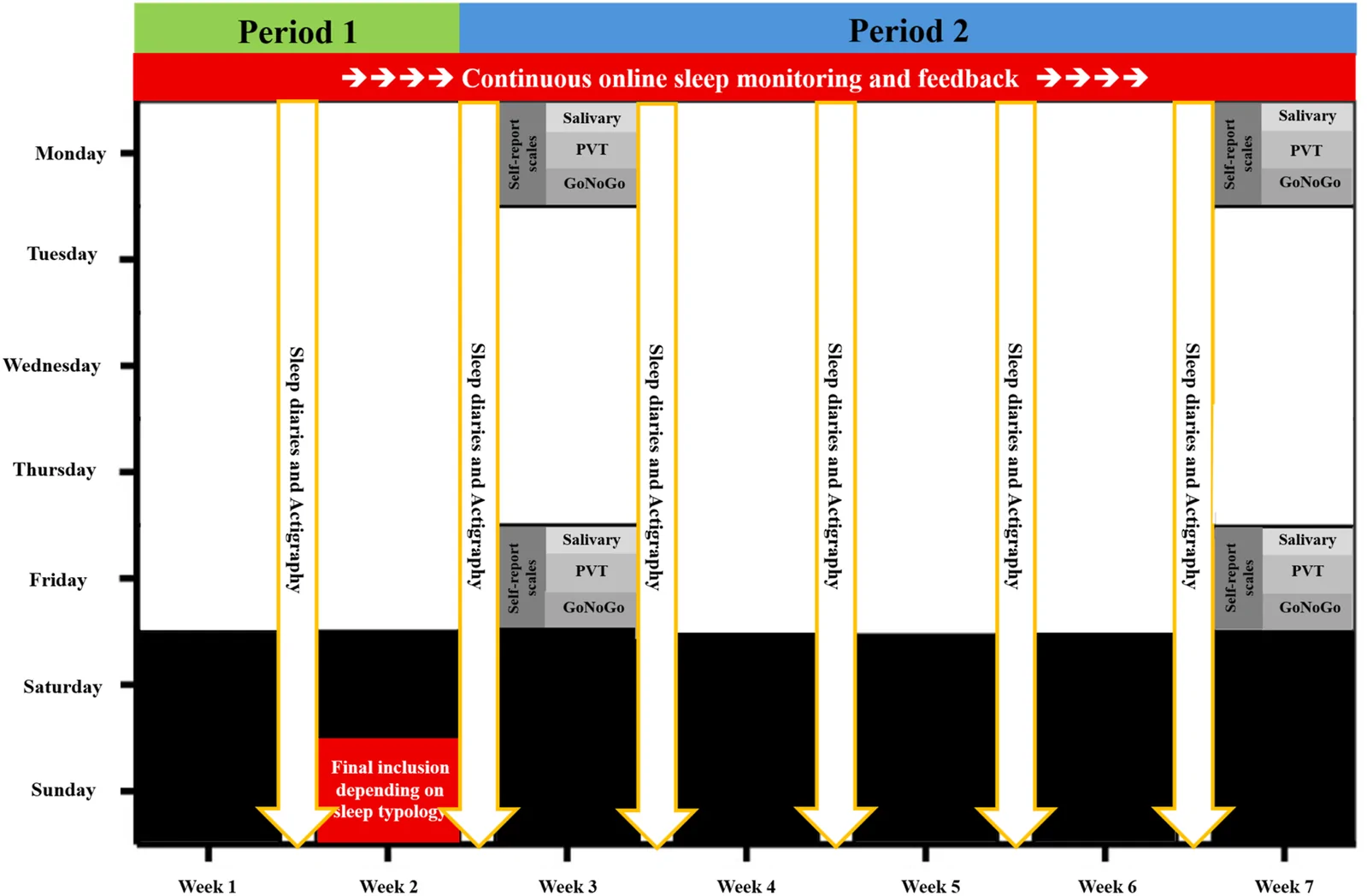
**Study protocol.** This protocol consists of a “*Period 1*” (P1: Weeks 1–2) followed by a “*Period 2*” (P2: Weeks 3–7). During P1, participants completed sleep diaries, and their sleep–wake patterns were continuously monitored using actigraphy recordings. Based on sleep typologies assessed at the end of the two-week baseline, only participants whose profiles aligned with one of the three experimental groups (Control, Debt, Debt + Nap) proceeded to the next phase of the protocol. Throughout P2, participants continued daily sleep monitoring (sleep diaries and actigraphy) and received continuous online sleep monitoring and feedback. On Monday and Friday of Weeks 3 and 7, participants attended laboratory sessions during which they completed a series of self-report scales, PVT, and Go-NoGo tasks, and provided salivary samples for hormonal and inflammatory analyzes.

### 2.3 Experimental measures

#### 2.3.1 Activity-based sleep monitoring (actigraphy)

Actigraphy and sleep diaries were used to record natural sleep-wake schedules. During the study period, all subjects were continuously monitored by an actigraphic device (MW8, CamTech; United Kingdom) mounted on a wristwatch and worn on the non-dominant wrist, coupled with a sleep diary that participants filled in to describe their sleep during the study period (Sadeh, 2011). Daytime sleep periods were detected only for the Sleep-Debt + Nap group; no other participants showed sleep episodes outside of the night-time in bed *(see Supplementary materials for more details*).

#### 2.3.2 Biochemical assays

Biological responses were evaluated through salivary biomarkers, including cortisol, IL-1β, IL-6, and brain-derived neurotrophic factor (BDNF). Participants were instructed to gently brush their teeth and rinse their mouths 60–90 min before sample collection (after breakfast) and to abstain from eating or drinking, except for water, until their arrival at the laboratory. Saliva samples were collected on Monday and Friday mornings at 9:00 a.m., under resting conditions, after participants had remained seated and calm for 15 min. Unstimulated saliva was collected in sterile 15-mL capped tubes and immediately centrifuged at 3000 g for 20 min at 4 °C. The supernatant was then aliquoted and stored at −80 °C until biochemical analyses (*see Supplementary materials for more details*).

#### 2.3.3 Psychomotor Vigilance Task (PVT)

We used a computerized version of the PC-PVT, adapted from Khitrov et al. (2014), to assess sustained attention under laboratory conditions. We extended the duration of the PC-PVT to a longer time duration (30 min), adapting the version previously employed by our team (for a review, see Arnal et al., 2015). Performance outcomes were expressed as mean Reaction Times (RTs, ms) and number of lapses. Valid RTs were defined as responses occurring between 150 and 500 ms. Lapses were defined as RTs exceeding 500 ms (Khitrov et al., 2014).

#### 2.3.4 Go-NoGo task

We used a computerized Go-NoGo paradigm (adapted from Donders, 1969; Sagaspe et al., 2012; Rabat et al., 2016) to assess response inhibition and sustained attention. We adapted the task to a longer time duration (30 min), that was previously used by our team (for a review, see Rabat et al., 2016). Performance outcomes were calculated as the number of commission errors (false responses on NoGo trials, reflecting inhibition failures) and omission errors (missed or delayed responses > 2 s on Go trials, reflecting vigilance lapses).

### 2.4 Data analysis

Statistical analyses were conducted using RStudio (v4.3.3, R Foundation for Statistical Computing) and Jamovi (v2.5.6, The Jamovi project, 2025, https://www.jamovi.org). Our analytical framework was structured to evaluate the specific interaction between chronic sleep debt and sustained mental effort. Linear mixed-effects models (LMMs; lme4, lmerTest) were applied to cognitive outcomes, using Participant ID as a random intercept. Fixed effects included Group (Control, Debt, Debt + Nap), Week (3 vs. 7), Day (Monday vs. Friday), and ToT (first 10 min vs. last 10 min of the 30-min task) to assess fatigability. To clarify temporal structure, “first” (or “initial”) and “last” refer to the internal segmentation of the 30-min cognitive tasks into 10-min bins: 0–10 min (first or initial) and 20–30 min (last). Models were evaluated via Type III ANOVA with Satterthwaite's approximation. Post-hoc comparisons were performed using Estimated Marginal Means (emmeans) with Holm correction. Non-parametric analyses utilized Kruskal–Wallis and Dunn's tests. Significance was set at *p* < 0.05. Results with p-values between 0.05 and 0.10 were considered trends and are reported to provide a more complete interpretation of potential effects, particularly given the relatively small sample sizes.

## 3 Results

### 3.1 Sleep patterns and protocol adherence

On weekdays (*from Sunday night to Thursday night)*, the Sleep-Debt group slept an average of 6 h and 7 min (±76 min), which is significantly less compared to the Control group (7 h and 26 min ± 41 min; *p* < 0.001; Table 1). This reduction in total sleep time during the week (Fig. 2) is associated with a noticeable extension of total sleep time during nights of the weekend (08h08 ± 01h07; +82 min; *p* < 0.001, Table 1). The Sleep-Debt + Nap group presented a comparable profile, with shorter nocturnal total sleep time on weeknights (6h14 min ± 74 min; *p* < 0.001; Fig. 2) combined with a partially offset by an average nap time of 53 min (±26 min). In the Sleep-Debt + Nap group, nap frequency averaged 2.2 ± 0.9 naps per weekday (range: 0.4–4.4). The substantial inter-individual variability was expected, as the ecological component of our protocol was specifically designed to preserve participants’ habitual and spontaneous napping behavior rather than imposing a fixed nap schedule. Consequently, nap frequency was allowed to vary naturally between participants. On weekends (*Friday and Saturday nights*), this group increased their total sleep time with an average of 7 h and 50 min (±58 min; *p* < 0.001). In contrast, the Control group maintained a stable total sleep time between weekdays and weekends (07h35 ± 00h37), with an average difference of 10 min (±16 min) in total sleep time and a stable bedtime and wake time (Table 1). No significant between-group differences in total sleep time were observed during weekends (Fig. 2B).

**Table 1:** Mean (± Standard Deviation) nocturnal and nap sleep durations (hours), bedtime, wake time and nap time during the weekdays and the weekend for the Control, Debt, and Debt + Nap groups. N.A = Non-Applicable.

	Control Group	Sleep-Debt Group	Sleep-Debt + Nap Group	
			Night	Nap
**Weekdays**	07h26 ± 00h41	06h07 ± 01h16	06h14 ± 01h14	00h53 ± 00h26
**Weekend**	07h35 ± 00h37	08h08 ± 01h07	07h50 ± 00h58	N.A
**Δ Weekend/Weekdays**	00h10 ± 00h16	01h22 ± 01h01	01h39 ± 00h41	N.A
Bedtime on weekdays	23h33 ± 0h46	00h51 ± 1h06	00h54 ± 1h05	
Wake time on weekdays	7h43 ± 0h53	7h50 ± 1h11	7h55 ± 1h07	
Bedtime on weekends	23h40 ± 0h57	00h52 ± 1h15	00h57 ± 1h18	
Wake time on weekends	8h09 ± 1h01	9h58 ± 1h43	9h17 ± 1h13	
Nap Time	N.A.	N.A.	Bedtime: 14h39 ± 0h46 Wake time: 15h24 ± 0h40

**Fig. 2:**
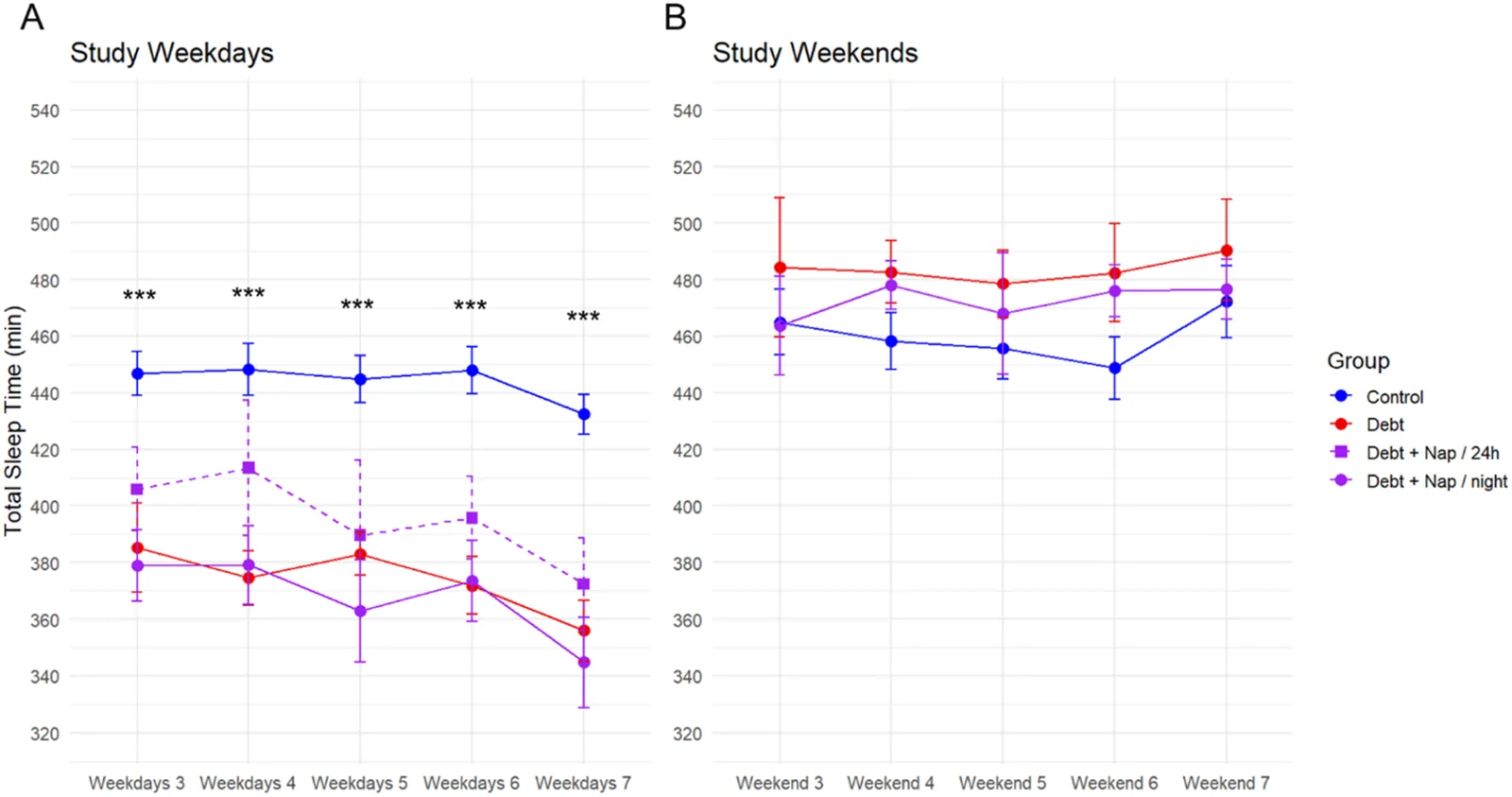
Evolution of total sleep time during the study protocol in each experimental group. **A)** Weekly changes in total sleep time (mean ± SEM) across the five study weeks (P2 period of the study) for the three experimental conditions: Control, Sleep-Debt, and Sleep-Debt + Nap, during weekdays (Sunday night to Thursday night). In the Sleep-Debt + Nap condition, total sleep time is reported both as nocturnal sleep duration only (Debt + Nap/night) and as the cumulative 24-h sleep duration including naps (Debt + Nap/24 h). Sleep duration remained stable across the five study weeks in all groups. Nocturnal sleep duration in the Sleep-Debt and Sleep-Debt + Nap/night conditions were significantly lower than in the Control group (***; p < 0.001). **B)** Weekly changes in total sleep time (mean ± SEM) across the five weekends (Friday and Saturday nights) for the three experimental groups. Participants in the Sleep-Debt and Sleep-Debt + Nap groups extended their sleep duration on weekends compared with weekdays, consistent with a pattern of catch-up sleep while the Control group maintained a stable sleep duration.

### 3.2 Impact of chronic sleep debt and benefit of napping on sustained attention (PVT)

#### 3.2.1 Reaction times (RTs)

We observed a significant effect of the Day (*F*(1, 83.7) = 5.51, *p* < 0.05). On Mondays, consistent with post weekend recovery states, only a main effect of ToT emerged (*F*(1, 84.66) = 22.06, *p* < 0.001), with no Group differences or interaction (all *p* values > 0.05). On Fridays, as a sleep debt accumulates progressively over the course of the Week, a significant effect of ToT (*F*(1, 82.93) = 19.29, *p* < 0.001) with a tendency of the Group effect (*F*(2, 28.62) = 3.24, *p* = 0.053) were found, with in addition, an interaction effect of these two factors (*F*(2, 82.93) = 4.06, *p* < 0.05). Post-hoc comparisons showed that RTs increased significantly from the first to the last 10-min for the Sleep-Debt group (*p* < 0.001) but not for the Control or Sleep-Debt + Nap groups (respectively, *p* > 0.05). Specifically, RTs were significantly higher in the Sleep-Debt group during the last 10 min compared to Controls (*p* < 0.01), whereas there was no significant difference during the first 10-min PVT (*p* > 0.67) (Fig. 3A). All post-hoc comparisons were adjusted using the Holm correction.

**Fig. 3:**
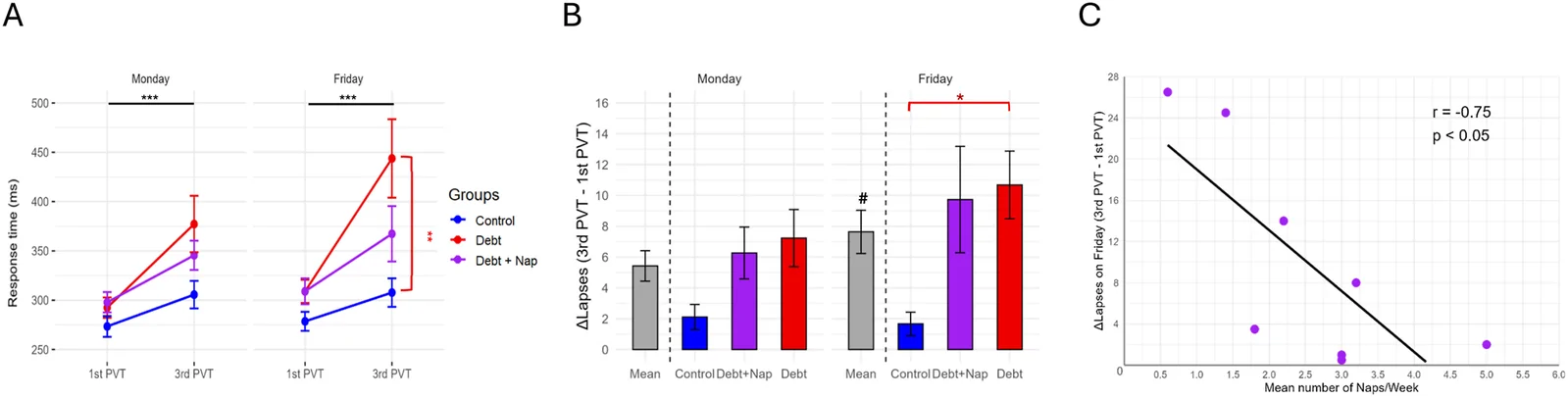
**Effects of sleep debt and naps on performance in the PVT. A)** Mean response time (± Standard Error of the Mean) during the first PVT and third PVT of the day, measured on Monday and Friday, for the Control (blue), Debt (red), and Debt + Nap (purple) groups. A time-on-task effect is observed, indicating a decline in vigilance throughout the task, particularly marked in the Debt group. On Fridays, response times for the Sleep Debt group are significantly higher than those for the Control group during the 3rd PVT. **B)** Changes in the number of lapses (ΔLapses = 3rd PVT – 1st PVT) on Monday and Friday for each group. Bars represent means ± SEM. A significant increase in the variation in the number of lapses was observed on Friday in the Debt group compared to the Control group. **C)** Negative correlation between the average number of weekly naps and the change in the number of lapses on Friday (r = −0.75, *p* < 0.05), suggesting a beneficial effect of naps on maintaining alertness after a period of sleep debt. *** (*p* < 0.001) indicates a significant difference between the 3rd and the 1st PVT period for all the participants independently of their group (Fig. 3A). # (*p* < 0.05) indicates a significant difference between the beginning (Monday) and the end (Friday) of the week independently of their group (Fig. 3B). ** (*p* < 0.01) and * (*p* < 0.05) indicate a significant difference between groups on Friday (Fig. 3A and B).

#### 3.2.2 Attentional lapses

We also observed a significant main effect of the Day (*F*(1, 84.1) = 4.41, p < 0.05). On Monday, results revealed only the effect of ToT (*F*(1, 84.7) = 21.56, *p* < 0.001), with no significant difference between groups or interactions (all *p* values > 0.05). On Friday sessions, we observed a significant effect of ToT (*F*(1, 82.9) = 25.54, *p* < 0.001) but with a tendency of the Group effect (*F*(2, 28.7) = 3.10, *p* = 0.060) associated with an interaction effect of these factors (F(2, 82.9) = 3.99, *p <* 0.05). Post-hoc comparisons showed that the number of lapses increased significantly during the PVT for both Sleep-Debt group (*p* < 0.001) and Sleep-Debt + Nap group (*p* < 0.001). These comparisons also revealed that the number significantly increased in both sleep-deprived groups during the last 10-min PVT compared to the Control group (Sleep-Debt: p < 0.01; Sleep-Debt + Nap: *p* < 0.05), whereas there were no such differences with the Control group during the first 10-min PVT (all *p* values > 0.1).

#### 3.2.3 Weekly evolution and napping

Fig. 3B illustrates the change in PVT lapses (ΔLapses, defined as the difference in lapses between the third and first PVT sessions) across the Week. To further examine the Day effect (*p* < 0.05), separate non-parametric analyses were performed. The analysis revealed a significant increase in lapses from the beginning to the end of the week (*p* = 0.05). No group differences were observed on Monday (*χ*^*2*^(2) = 2.36, *p* > 0.05), whereas a significant Group effect emerged on Friday (*χ*^*2*^(2) = 8.67, *p* < 0.05) where Controls had fewer lapses than the Sleep-Debt group (*p* < 0.05), with a trend toward a difference between the Control and Sleep-Debt + Nap groups (*p* = 0.065). On Friday, the Sleep-Debt + Nap group displayed intermediate performance but marked inter-individual variability. Given the naturalistic design allowing variable nap adherence, we examined whether nap frequency explained this dispersion. Nap frequency was negatively correlated with Friday ΔLapses (*r* = −0.75, *p* < 0.05; Fig. 3C), indicating that more frequent napping was associated with better maintenance of vigilance (smaller lapses increases).

### 3.3 Cumulative effect of sleep debt on response inhibition

#### 3.3.1 Commission rates

A significant main effect of the Day was observed (*F*(1, 86.2) = 14.75, *p* < 0.001). On Monday sessions, results revealed a ToT effect (*F*(1, 87.03) = 10.77, *p* < 0.001), neither the group effect nor the interaction reached significance (all *p* values > 0.05). Following the significant main effect of ToT, comparisons between groups were conducted independently for each session. During the first 10-min of the Go-NoGo task (GNG), a significant effect of the Group was observed (*χ*^*2*^(2) = 6.72, *p* < 0.05), with only a difference between the Sleep-Debt group and the Control group (*p* < 0.05). In opposition, during the last 10-min of the GNG, no significant difference was observed between the groups (*χ*^*2*^(2) = 4.81, *p* > 0.05). On Friday, matching with the peak of accumulated sleep debt, the effect of ToT remained significant (*F*(1, 85.8) = 10.28, *p* < 0.01). However, in contrast to the finding observed on Monday, group differences demonstrated heightened persistence (Fig. both *p* < 0.05 for the first and last 10 min). The Sleep-Debt group exhibited significantly elevated commission rates in comparison to the Control group during both the initial and final 10-min (*p* < 0.05). A trend toward a difference between the Sleep-Debt and Sleep-Debt + Nap groups was also found for the initial 10-min (*p* = 0.06).

#### 3.3.2 Omission rates

A notable principal effect of the Day was noted for omission rates (*F*(1, 85.6) = 5.27, *p* < 0.05). On Monday, while ToT again showed a measurable influence on omission error rate (*F*(1, 86.9) = 4.14, *p* < 0.05), no Group effect or interactions were exposed (all *p* values > 0.05). On Friday sessions, the impact of ToT intensified (F(1, 87.3) = 11.54, *p* < 0.01), with no Group effect (*F*(2, 30.1) = 1.67, *p* > 0.1). Although a trend toward an interaction between these factors was observed (*F*(2, 87.3) = 2.81, *p* = 0.065). Planned contrasts revealed significant increases in omission rates from the start to the end of the GNG task for both sleep-deprived groups (Sleep-Debt group: 1.27 ± 2.93 → 5.66 ± 10.32; *p* < 0.01); Sleep-Debt + Nap: 1.18 ± 1.36 → 5.71 ± 9.12; *p* < 0.01), whereas the Control group remained stable (*p* > 0.10). These contrasts showed notably more omissions in the Sleep-Debt group than Controls in the last 10-min (5.66 ± 10.32 vs. 0.27 ± 0.36; *p* < 0.05). In contrast, the Sleep-Debt + Nap group did not differ significantly from Controls (*p* > 0.05), suggesting that regular napping may buffer the cumulative impact of sleep debt on selective attention during sustained mental exertion.

#### 3.3.3 Weekly progression of omission error variability (ΔOmissions)

As illustrated in Fig. 4B, the group-specific change in omission rates is expressed as the variation between the third and first GNG (ΔOmissions) over testing days. Analyses revealed a significant effect of Day (*p* < 0.05), with no Group effect (*p* > 0.10), and a trend toward a Day × Group interaction (*p* = 0.09). A significant increase in the prevalence of ΔOmissions was observed from Monday to Friday across the entire cohort (*p* < 0.05). While no significant differences were found between groups on Monday (*p* > 0.10), a significant Group difference emerged on Friday (*p* < 0.05). Specifically, the Control group demonstrated a significantly lower prevalence of ΔOmissions in comparison to the Sleep-Debt group (*p* < 0.05) and the Sleep-Debt + Nap groups (trend at *p* = 0.068).

**Fig. 4:**
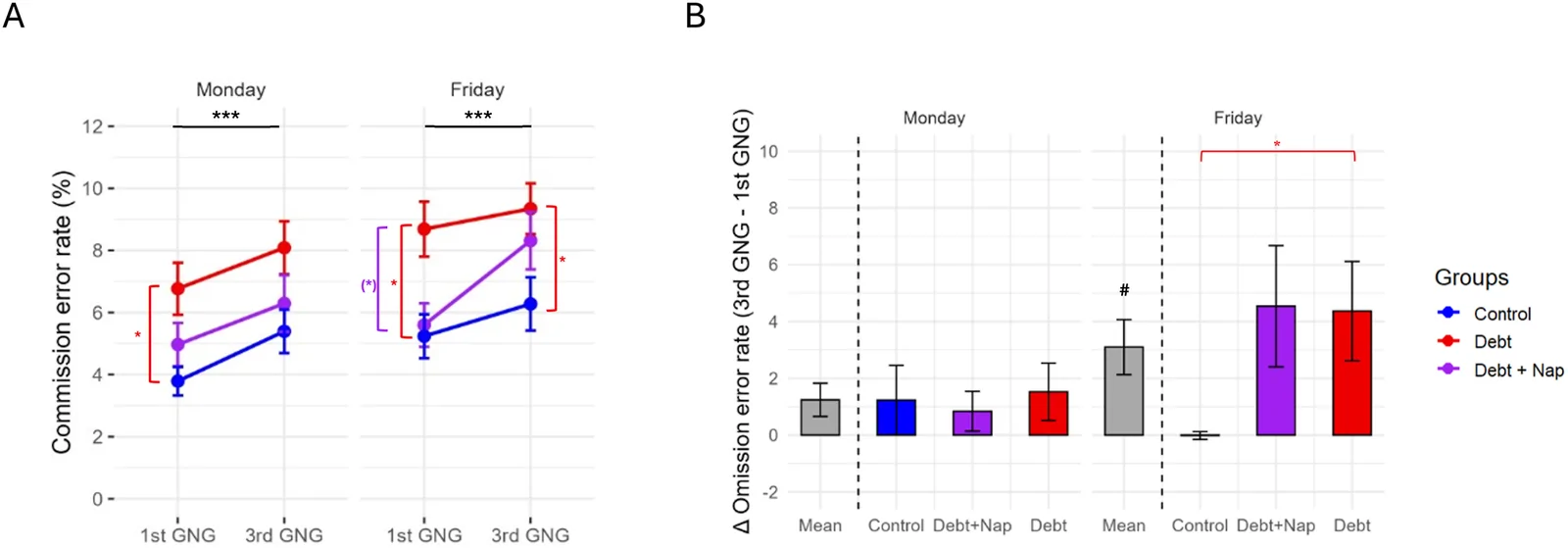
**Effects of sleep debt and naps on different error rates in the Go-NoGo. A)** Commission error rate (±SEM) during the 1st and 3rd Go/No-Go (GNG) tests measured on Monday and Friday for the Control, Debt, and Debt + Nap groups. The Debt group exhibited higher commission error rates than the Control group at the 1st GNG on both Monday and Friday and showed a trend toward higher error rates compared to the Debt + Nap group on Friday (*p* < 0.05). At the 3rd GNG on Friday, the Debt group also showed significantly higher error rates than the Control group (*p* < 0.05). **B)** Changes in omission error rate (ΔOmission = 3rd GNG – 1st GNG) on Monday and Friday for each group. Bars represent means ± SEM. A significant increase in the variation in the omission error rate was observed on Friday in the Debt group compared to the Control group (*p* < 0.05). *** (*p* < 0.001) indicates a statistical difference between the 3rd and the 1st GNG period for all the participants independently of their group (Fig. 4A). # (p < 0.05) indicates a statistical difference between the beginning (Monday) and the end (Friday) of the week independently of their group (Fig. 4B). * (*p* < 0.05) and ((*) *p* = 0.06) respectively indicate a significant and a trend difference between groups on Monday and Friday (Fig. 4A and B).

### 3.4 Biological stress, inflammatory, and neurotrophic responses to chronic sleep debt with or without napping

#### 3.4.1 Cortisol

We reported a main effect of Week and a significant Group × Week interaction (all *p* < 0.05) for morning cortisol levels. After three weeks of follow-up, the circadian cortisol profile remained largely stable across all three groups, as reflected by comparable cortisol levels during the morning surge window (all *p* > 0.05 at 09:00 a.m.; Fig. 5A). In contrast, after seven weeks, accumulated sleep debt was associated with a blunted circadian cortisol rhythm, evidenced by a dampened morning cortisol response on Friday in the Sleep-Debt group (main Group effect *p* = 0.002; main Day effect *p* < 0.001; *p* > 0.10 for Group × Day interaction at 09: 00a.m; Fig. 5B). Pairwise comparisons for group differences revealed significantly lower cortisol levels on Friday in sleep-deprived participants compared to Controls (*p* < 0.05). Notably, the pronounced reduction in cortisol observed on Friday morning—the day corresponding to the peak of accumulated sleep debt—was no longer present on Monday, after a weekend of recovery sleep (*p* < 0.01). Importantly, participants in the chronic sleep-deprived group who engaged in daily napping did not exhibit blunting of cortisol levels during the morning period of maximal cortisol secretion at 09:00 a.m. after seven weeks (*p* > 0.10 vs. Controls and *p* < 0.01 vs. Sleep-Debt group). Specifically, analyses between Week 3 and 7 further revealed a significant reduction—by approximately 50%—in morning cortisol levels at 09: 00a.m on Friday morning between Week 3 and Week 7 in the Sleep-Debt group (*p* < 0.05 for main effect of Week; *p* > 0.10 for main effect of Group; *p* < 0.05 for Group × Week interaction; *p* < 0.05 for post hoc pairwise comparison between Week 3 and Week 7 in Sleep-Debt group; Fig. 5C).

**Fig. 5:**
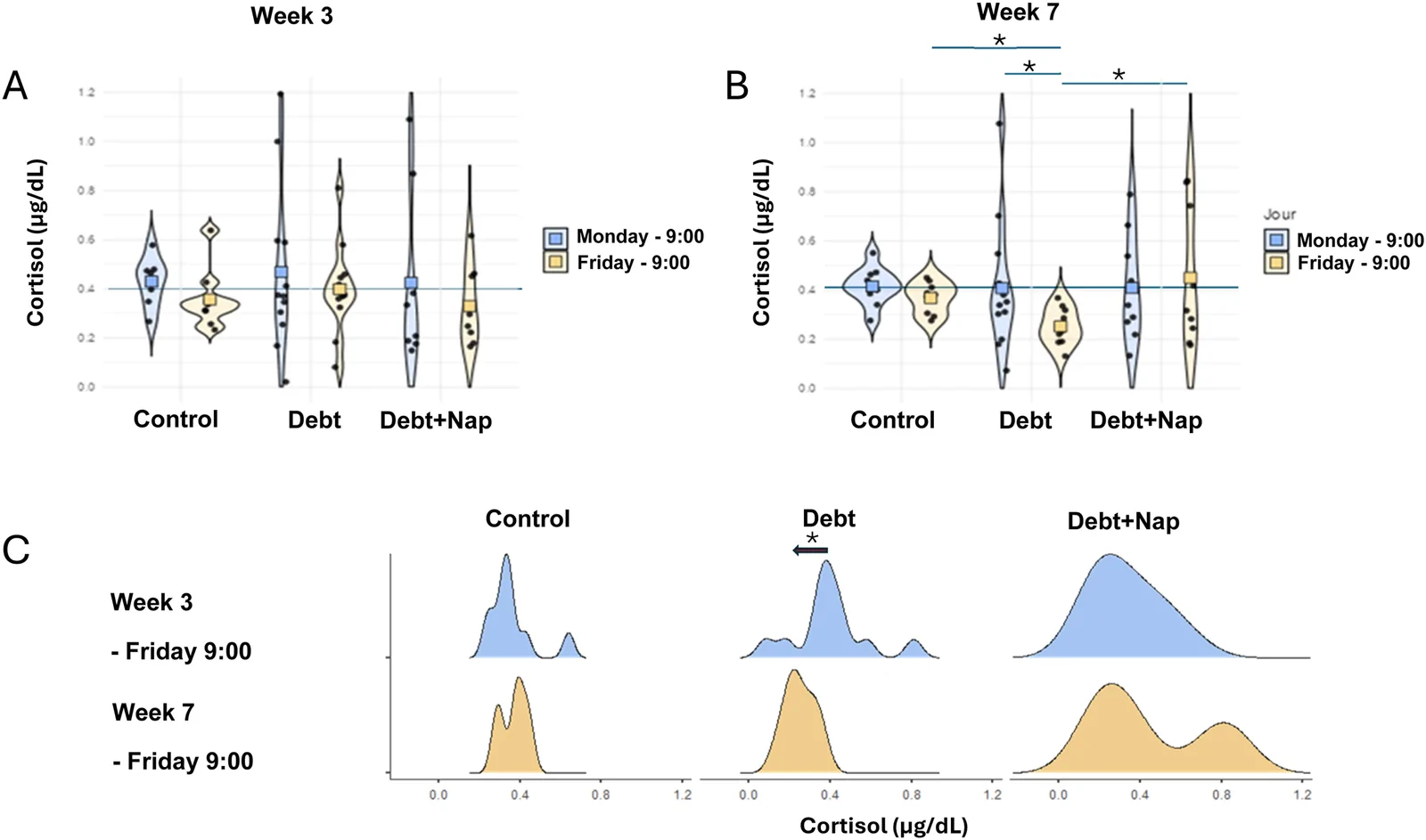
**Effects of sleep debt and naps on morning cortisol levels during weeks 3 and 7. A)** Morning cortisol concentration (μg/dL) measured at 09:00 on Monday and Friday during Week 3 for the Control, Debt, and Debt + Nap groups. No significant differences were observed between groups or days for Week 3. **B)** Cortisol levels measured at 09:00 on Monday and Friday during Week 7. The Debt group showed a significant decrease in cortisol from Monday to Friday (*p* < 0.05). On Friday, cortisol levels were significantly lower in the Debt group compared to both the Control and Debt + Nap groups (*p* < 0.05, *p* < 0.01), indicating a blunted morning cortisol response following chronic sleep restriction. **C)** Density plots illustrate the distribution of cortisol concentrations at 09:00 on Friday for weeks 3 and 7 in each group. The Debt group exhibited a downward shift in cortisol distribution at Week 7 compared to Week 3 (*p* < 0.05), while the Control and Debt + Nap groups maintained stable profiles over time.

#### 3.4.2 Interleukin-1β and interleukin-6

A main Group effect was reported for IL-1β and IL-6 (*p* < 0.001 and *p* < 0.05 respectively) but no Week effect or Group × Week interaction was noted (all *p* > 0.10) preventing us from performing a Week-by-Week discriminator analysis. With respect to the difference between the groups, Monday levels of IL-1β were significantly higher in the Sleep-Debt group than in the Control group (Group effect *p* < 0.001, no Day or Group × Day interaction effects, pairwise comparisons Sleep-Debt vs. Control, *p* < 0.05). On Friday, this time, with the accumulation of sleep debt over the course of the Week, IL-1β levels were significantly higher than Controls (*p* < 0.001) but also than Sleep-Debt + Nap group (*p* < 0.01; Fig. 6A). Regarding Monday levels of IL-6, sleep-deprived participants display higher concentrations than both Control and Sleep-Debt + Nap groups (Group effect *p* < 0.05, no Day or Group × Day interaction effects, pairwise comparisons, Sleep-Debt vs. Control and Sleep-Debt + Nap groups, both *p* < 0.05; Fig. 6B).

**Fig. 6:**
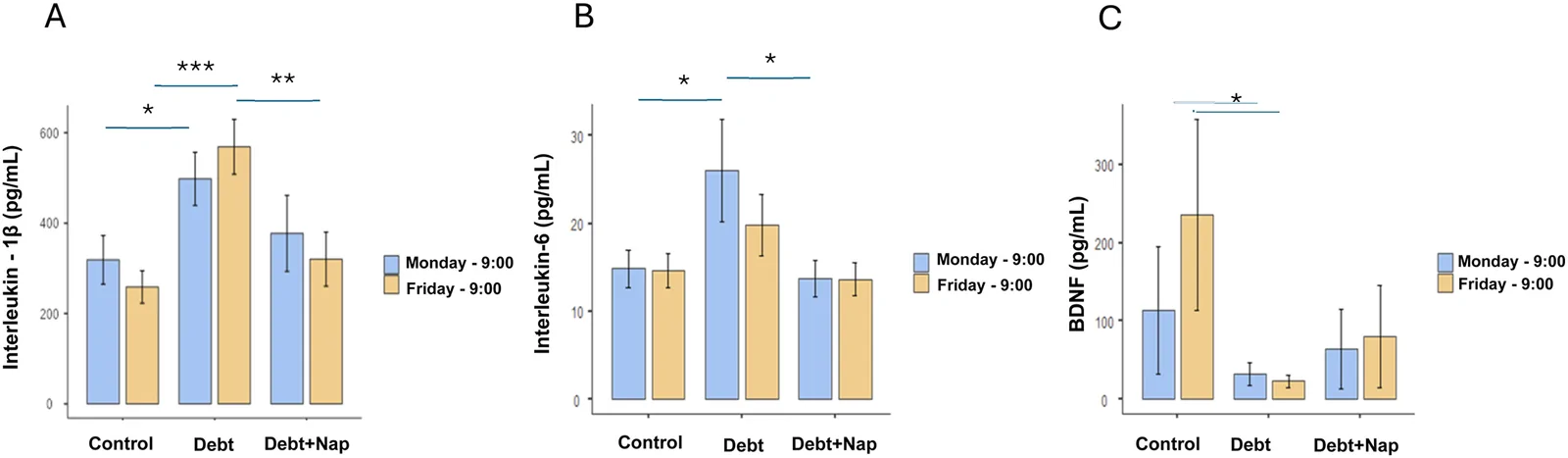
**Effects of sleep debt and naps on inflammatory and neurotrophic markers measured on Monday and Friday mornings. A)** Interleukin-1β (IL-1β) levels (pg/mL) were significantly higher in the Debt group compared to the Control group on both days (respectively *p* < 0.05, *p* < 0.001), and higher than in the Debt + Nap group on Friday (*p* < 0.01). **B)** Interleukin-6 (IL-6) levels (pg/mL) were significatively higher in the Debt group compared to both Control and Debt + Nap groups on Monday (*p* < 0.05). **C)** Brain-Derived Neurotrophic Factor (BDNF) levels (pg/mL) were lower in the Debt group compared to the Control group (*p* < 0.05). These findings indicate that sleep debt increases pro-inflammatory cytokine levels and reduces neurotrophic support, whereas napping appears to attenuate these effects.

#### 3.4.3 Salivary IL-1β levels predict ToT decline in psychomotor vigilance under sleep deprivation

Among the various biomolecules of interest quantified in this study, IL-1β emerged as a specific biological predictor of the ToT decline. We observed a robust positive correlation between morning IL-1β levels and the magnitude of performance degradation during the 30-min PVT. Notably, IL-1β levels were positively and significantly correlated with the ToT decline in performance on the PVT. Specifically, higher concentrations of this pro-inflammatory cytokine significantly predicted both the increase rate of RT (ΔRT; r = 0.42, p = 0.024; Fig. 7A) and the accumulation rate of lapses (ΔLapses; r = 0.48, p = 0.007; Fig. 7B). These findings suggest that higher circulating levels of IL-1β may be associated with a greater deterioration in sustained attention capabilities over a prolonged cognitive engagement combined with or without a sleep debt. Conversely, lower levels of IL-1β appear to be linked to a better ability to maintain performance throughout the task, as reflected in both faster RTs and fewer attentional lapses.

**Fig. 7:**
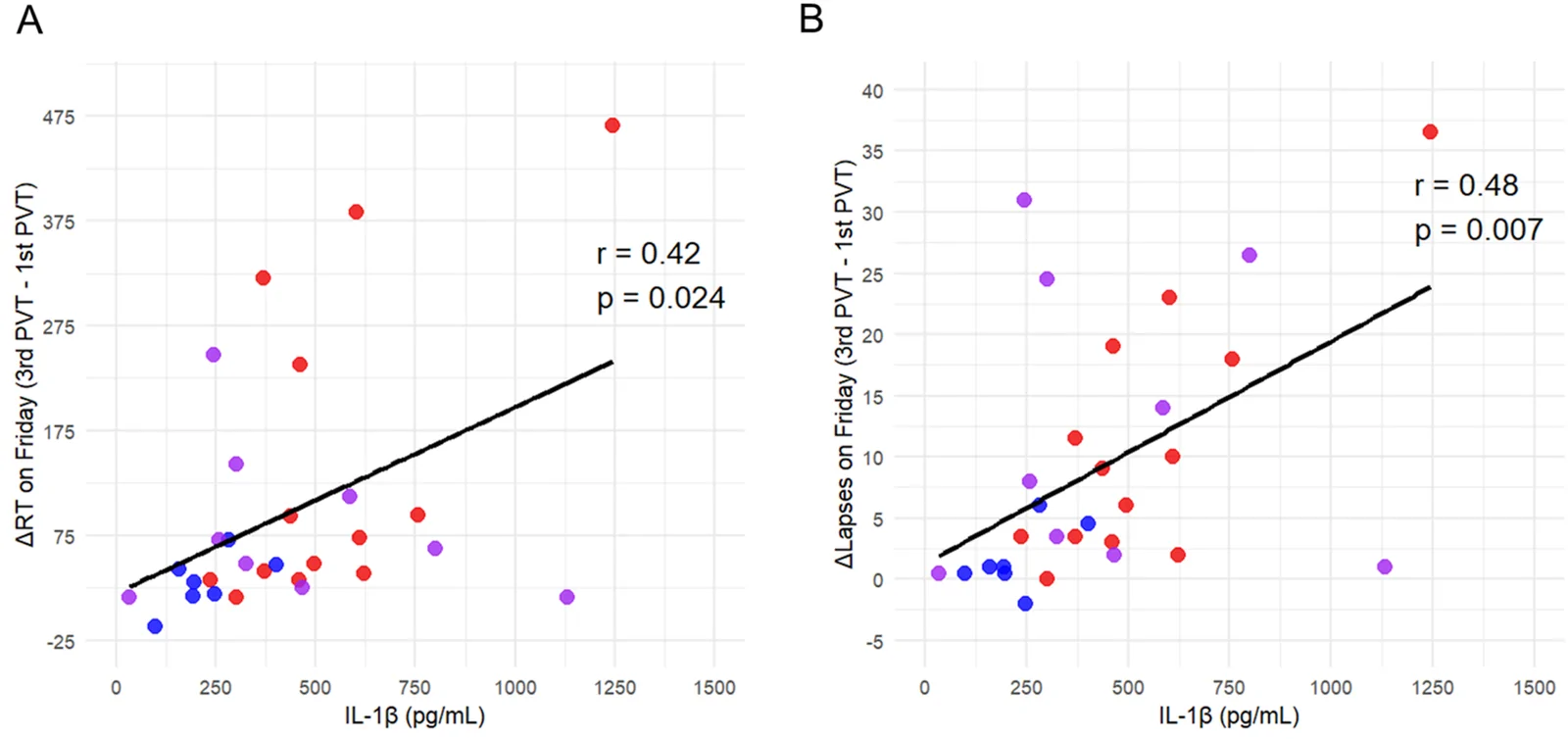
**Correlations between salivary interleukin-1β (pg/mL) and PVT performance on Friday for all subjects (N = 32). A)** Positive correlation between IL-1β concentrations and changes in mean response time (ΔRT = 3rd PVT – 1st PVT) on Friday (r = 0.42, *p* = 0.024). Blue dots represent the Control group, red dots represent the Sleep-Debt group and purple dots represent the Sleep-Debt + Nap group. **B)** Positive correlation between IL-1β concentrations and the variation in the number of lapses (ΔLapses = 3rd PVT – 1st PVT) on Friday (r = 0.48, *p* = 0.007). High levels of IL-1β in saliva have been associated with greater impairment in alertness, suggesting a link between peripheral inflammatory activity and attentional deficits following chronic sleep debt. Blue dots represent the Control group, red dots represent the Sleep-Debt group and purple dots represent the Sleep-Debt + Nap group.

#### 3.4.4 Brain-derived neurotrophic factor

We also measured morning concentrations of the neurotrophic factor BDNF in all study participants during experimental Weeks 3 and 7. A main Group effect was reported for BDNF levels (*p* < 0.05) but no Week effect or Group × Week interaction was noted (all *p* > 0.1) not allowing for a weekly discriminant analysis to be performed. In terms of group differences, on Friday, the Sleep-Debt group exhibited markedly lower BDNF concentrations than the Control group (Group effect *p* < 0.05, no Day or Group × Day interaction effects, pairwise comparisons Sleep-Debt vs. Control, *p* < 0.05; Fig. 6C).

## 4 Discussion

Our aim was to propose and to test novel hypotheses on mental fatigue by examining the interaction between physiological constraints associated with chronic sleep debt and prolonged cognitive demand in healthy individuals. To this end, we adopted a more physiologically grounded approach under ecologically valid conditions. We consider real-world research as a complementary strategy that can inform, enrich, and inspire laboratory investigations and conversely, laboratory findings can guide and refine field studies.

Our present study revealed that Time on Task (ToT) instigates cognitive fatigue in both sustained attention and inhibition, though chronic sleep debt exerts differential effects on these processes. Utilizing a prolonged PVT, we demonstrated that chronic sleep debt significantly exacerbates ToT-related declines in sustained attention, more than in executive inhibition. In contrast, the regular practice of napping during the week effectively mitigated sustained attention deficits related to both ToT and sleep debt (RTs and lapses). Nevertheless, inhibitory failures (commission errors with the Go-NoGo) exhibited an increase with ToT, demonstrating an additive, stable sleep debt effect regardless of the day of the week. From a biological standpoint, a state of chronic sleep debt was associated with a diminished cortisol rhythm and heightened pro-inflammatory cytokine levels, such as IL-1β and IL-6. These findings suggest the possibility of HPA axis dysregulation and the presence of low-grade systemic inflammation. Importantly, salivary IL-1β levels significantly correlated with sustained attention deficits. In conjunction with modified neurobiological markers (e.g., BDNF), this immuno-inflammatory state provides a physiological explanation regarding sustained attention deficits related to both ToT and chronic sleep debt.

A notable strength of our design lies in its dual-scale analysis. While cognitive performance was assessed daily (Monday vs. Friday) to capture the acute interaction between weekend recovery and cumulative sleep debt pressure, salivary biomarkers were monitored longitudinally. This distinction is grounded in physiological reality: cognitive functions are highly sensitive to immediate homeostatic sleep pressure and circadian fluctuations, whereas inflammatory and neuroendocrine markers reflect a more stable, systemic adaptation to chronic sleep restriction. This multi-level monitoring facilitates the distinction between the “acute” fatigue induced by the task and the “chronic” biological terrain upon which this fatigue develops.

### 4.1 Cognitive fatigue: an implication of time on task but a differential effect of sleep debt

Our study contributes by combining ecological and laboratory assessments to demonstrate an interactive effect between prolonged cognitive demand (ToT) and moderate but chronic sleep debt (time-on-week). This interaction selectively amplified sustained attention failures, leaving executive inhibitory capacities largely unaffected.

The increased fatigability of sustained attention across the week is consistent with prior laboratory and field studies showing a progressive rise in sleepiness and attention deficits during multi-day sleep restriction (Belenky et al., 2003; Van Dongen et al., 2003; for a review, see Lim and Dinges, 2008). Our findings are also consistent with previous work demonstrating the deleterious effects of ToT on sustained attention, even in studies using shorter PVT durations that are arguably less representative of real-world working conditions (Guo et al., 2016; Lim et al., 2010; Quiquempoix et al., 2022). They also align with extensive evidence showing that chronic sleep debt impairs sustained attention (Dinges et al., 1997; Belenky et al., 2003; Rabat et al., 2016). Furthermore, our results support the hypothesis that sustained attention processes are more vulnerable to sleep loss than executive functions, both under total sleep deprivation (TSD; Quiquempoix et al., 2022; Drummond et al., 2005; Arnal et al., 2015; Rabat et al., 2019) and partial sleep restriction (Rabat et al., 2016; Lim and Dinges, 2008; Lo et al., 2012). Supporting this, Lo et al. (2012) reported a modest effect size of one week of sleep history (sleep restriction compared to controls) on working memory deficits related to TSD, whereas substantially larger effects were observed for both sleepiness and sustained attention.

Since total sleep deprivation (TSD) is associated with significant and specific reductions of the metabolism of fronto-parietal networks (Thomas et al., 2000; Ma et al., 2015) dedicated to sustained attention (Drummond et al., 2005; Lim and Dinges, 2008), chronic moderate sleep debt logically interferes with the same networks. Meta-analytic work on TSD confirms that fronto-parietal attention networks are among the most affected regions (Ma et al., 2015) justifying the hypothesis that sustained attention is more vulnerable to sleep debt compared to executive ones such as inhibition; (Lo et al., 2012; Rabat et al., 2016). Moreover, these processes engage different frontal brain regions, with an activation of the right dorsolateral prefrontal cortex for sustained attention (Culham et al., 2001; Yamasaki et al., 2002) and the right ventrolateral part for inhibition (Aron et al., 2004). Furthermore, some individuals maintained better inhibitory performance, under TSD, through compensatory activation in the right ventrolateral prefrontal cortex and insula (Drummond et al., 2006; Chuah et al., 2006). Indeed, better performance at higher cognitive loads for executive functions (i.e. working memory) after 35 h of TSD, is related to increased cerebral responses within parieto-frontal networks (Drummond et al., 2004, 2005; Lythe et al., 2012).

In agreement with Frenda and Fenn (2016), we argue that a moderate chronic sleep debt primarily impairs the ability of individuals to be alert and to sustain their attention, while allowing higher executive functions to operate under compensatory mechanisms (Drummond et al., 2006; Drummond et al., 2005). This could explain why executive deficits (i.e. inhibition) in this study are mainly related to ToT. This interpretation is further supported by converging evidence from pharmacological studies showing that stimulants (i.e., caffeine, modafinil, and dextroamphetamine) can effectively mitigate sustained attention deficits induced by TSD, whereas their effects on executive functioning remain limited (Wesensten et al., 2002, 2005; Killgore et al., 2008). Similarly, countermeasures such as sleep extension and regular physical activity were beneficial for sleepiness and sustained attention deficits but not for executive functions (inhibition and working memory) under conditions of TSD (Arnal et al., 2015; Rabat et al., 2019; Sauvet et al., 2020) or under chronic sleep restriction (Rupp et al., 2009).

### 4.2 Cognitive fatigue: a partial benefit of chronic daytime naps

Our findings indicate that regular early-to-mid afternoon napping partially mitigates morning sustained attention failures under moderate, chronic sleep restriction, supporting the restorative hypothesis of daytime sleep. Notably, this benefit appears long-lasting, with effects evident in next-morning assessments persisting over 24 h. These results align with evidence that regular naps sustain alertness and attenuate cognitive decrements due to ToT (Gillberg et al., 1996; Faraut et al., 2017; Leong et al., 2022; Dutheil et al., 2021; Lo et al., 2017). We hypothesized that napping reduces sleep homeostatic pressure and extracellular adenosine accumulation, counteracting its inhibitory effect on fronto-parietal networks involved in sustained attention processes. Besides, the effect of napping on inhibitory control (Go-NoGo task) appeared limited. Commission errors, reflecting impulsive responses, significantly increased with ToT across all groups and were consistently higher in the Sleep-Debt group compared to Controls. Although participants in the Sleep-Debt + Nap group tended to show slightly fewer commission errors than those experiencing chronic sleep debt, the effect size was small (Cohen's d = 0.28). Thus, while a potential beneficial effect of napping on inhibitory control cannot be excluded, these findings should be interpreted cautiously given the limited sample size of the Sleep-Debt + Nap group (n = 9). This mechanism may explain the enhancement of sustained attention performance, as reflected by improved PVT outcomes following napping. This interpretation is consistent with Lo et al. (2017), who demonstrated that napping mitigates the decline in sustained attention and the slowing of RTs typically induced by prior sleep restriction. In contrast, for omission errors, reflecting attentional disengagement, the effect of napping was more pronounced. Both sleep-deprived groups exhibited a significant increase in omissions from the first to the last Go-NoGo block, but this increase was considerably greater in the Sleep-Debt group compared to Controls, whereas the Sleep-Debt + Nap group did not differ significantly from the Control group. This pattern confirms that napping partially protected sustained attentional processes compromised by chronic sleep debt. Importantly, nap frequency across the week was negatively correlated with the increase in lapses related to ToT (r = −0.75, *p* < 0.05), suggesting that regular naps mitigated sustained attention failures. A closer examination of the Sleep-Debt + Nap group revealed substantial inter-individual napping practice variability due to the ecological protocol. We anticipated that varying adherence to the ecological napping protocol—given that participants were free to nap whenever their daily schedules allowed—would introduce substantial inter-individual variability in nap frequency and characteristics (timing, duration, and sleep quality), potentially explaining the differences observed in sustained attention performance. Participants who napped more frequently exhibited performances comparable to Controls, whereas those who napped less frequently showed behavioral profiles similar to the Sleep-Debt group. This clear dose-dependent effect suggests that daytime sleep benefits depend not only on its availability but on regularity and cumulative frequency, supporting a behavioral “dose–response” relationship between nap frequency and sustained attention (Lo et al., 2017, 2022).

Taken together, these findings suggest that napping benefits cognitive domains differently, with stronger effects on vigilance and sustained attention than on executive inhibitory control (Rabat et al., 2019; Dutheil et al., 2021; Sauvet et al., 2020). This dissociation may reflect the rapid recovery of alertness networks during short sleep episodes, whereas prefrontal executive functions may require longer or more consolidated sleep (Ma et al., 2015; Drummond et al., 2006). Consistent with neuroimaging evidence, prefrontal hypoactivation during sleep loss appears to affect vigilance and attentional stability before inhibitory control (Chuah and Chee, 2008; Ma et al., 2015; Krause et al., 2017). Differences in adenosine-related sleep pressure across cortical regions may further contribute to this selective vulnerability (Elmenhorst et al., 2007). Finally, the additive decline observed across the week suggests a gradual deterioration rather than a threshold effect. The intermediate performance of the Sleep-Debt + Nap group further indicates that regular daytime naps may mitigate attentional decline by supporting functional connectivity within frontoparietal attention networks.

### 4.3 Cognitive fatigue: potential mechanisms linking sleep debt to stress and immuno-inflammatory dysregulation

Across all three groups (Control, Sleep-Debt, and Sleep-Debt + Nap), IL-1β concentrations were positively associated with declines in sustained attention, reflected by increased reaction times (RTs) and accumulated lapses (Fig. 7). Higher IL-1β concentrations significantly predicted both outcomes. Similarly, Lasselin et al. (2016) showed that low-grade inflammation contributes to attentional deficits. Our findings therefore suggest that increased proinflammatory signaling may be associated with attentional impairment under chronic sleep debt.

IL-1β and tumor necrosis factor alpha (TNF-α) are key cytokines involved in sleep regulation, with both cytokines and their receptors expressed in brain regions involved in sleep–wake regulation (Imeri and Opp, 2009; Breder et al., 1988, 1993). Animal studies show that IL-1β promotes non-rapid eye movement (NREM) sleep, while blocking IL-1 receptors in humans reduces subjective sleepiness (Krueger et al., 2001; Lehrskov et al., 2018). IL-1β may therefore contribute to both physiological sleep regulation and sleep alterations induced by sleep restriction (Irwin, 2002; Irwin and Opp, 2017; Rowe and Griesbach, 2022).

Cytokines may also contribute to local sleep processes, whereby specific brain regions transiently exhibit sleep-like activity independently of global arousal (Krueger et al., 2011; Andrillon and Oudiette, 2023). Astrocytes and microglia may participate in these processes through inflammatory signaling and modulation of BDNF, potentially amplified by elevated adenosine levels (Carvalhas-Almeida et al., 2023). Regional differences in cytokine expression and receptor sensitivity may further contribute to these effects (Silverman et al., 2014).

Cytokine-driven local sleep processes may thus represent a mechanism linking inflammation to cognitive impairment during sleep loss (Besedovsky et al., 2019; Irwin, 2019). The reduction in BDNF observed under sleep debt may indicate altered neuroplasticity, whereas its partial preservation in the Sleep-Debt + Nap group may be consistent with a potential restorative effect of napping. However, these findings do not establish a causal relationship.

Finally, sleep loss may disrupt immune-inflammatory regulation through interactions between the sympathetic nervous system and the hypothalamic–pituitary–adrenal (HPA) axis. While catecholamines can promote proinflammatory signaling, cortisol normally provides anti-inflammatory feedback (McEwen, 2006; Charmandari et al., 2005). The reduced morning cortisol levels observed under sleep debt may therefore reflect altered HPA-axis regulation, consistent with our previous finding of decreased morning salivary cortisol following severe sleep restriction (Faraut et al., 2020).

### 4.4 Study limitations, conclusion and future perspectives

While these findings provide novel insights into real-world sleep debt, several methodological considerations warrant discussion. First, although the ecological nature of the study is a primary strength, the lack of polysomnography remains a limitation. Actigraphy, while validated for longitudinal monitoring, does not allow detailed analysis of sleep architecture or objective quantification of sleep stages. Similarly, the absence of EEG recording during cognitive testing prevented us from characterizing the neurophysiological changes—such as shifts in theta power—associated with the interaction between chronic sleep debt and ToT effects. Future studies using mobile EEG or longer cognitive tasks (e.g., 45 min) could elucidate whether chronic sleep debt induces specific topographic brain activity patterns compared to acute sleep loss. Second, group stratification was based on participants' natural sleep typologies and habitual napping behaviors. While this design preserved high ecological validity, sample sizes per group are relatively small, which may have limited statistical power to detect subtle interactions, particularly for executive functions. A limitation of the present study is the relatively small and unbalanced final sample (n = 32). To account for these group sizes and the longitudinal repeated-measures design, we used linear and generalized linear mixed-effects models with appropriate degrees-of-freedom estimation. Each participant contributed four strictly standardized within-subject observations (Monday and Friday measurements repeated at two time points), which were explicitly accounted for in the models. In addition, a larger cohort (n = 75) was followed during the initial two-week phase (P1), whereas the final sample reflected the substantial burden of the subsequent five-week experimental protocol and the need to rigorously confirm chronic sleep debt. Finally, the association between PVT performance (ΔRTs and ΔLapses) and IL-1β, initially observed in the Sleep-Debt subgroup (n = 13), remained significant and of comparable strength when re-evaluated in the full sample (n = 32; Fig. 7), supporting the robustness of this finding. Nevertheless, the limited and unbalanced sample size, particularly for interaction and correlation analyses, should be considered when interpreting the findings.

Furthermore, as nap timing and frequency of these naps were not experimentally standardized, the resulting inter-individual variability requires cautious interpretation of the napping “dose-response” effect. Finally, a notable limitation is the lack of control for physiological variables, particularly sex differences and menstrual cycle phases. The skewed sex ratio and the lack of systematic assessment of menstrual cycle phase or oral contraceptive use may have introduced potential confounding effects, as salivary biomarkers, including cortisol, IL-1β, and IL-6, may be sensitive to fluctuations in sex hormones (Konecna et al., 2000; Klusmann et al., 2023). Future longitudinal studies should account for these hormonal factors to better isolate the impact of chronic sleep debt and napping on neuro-immune and neurotrophic responses.

Overall, this study provides compelling evidence that chronic sleep debt, under real-world conditions, acts as a potent stressor that accelerates the onset of cognitive fatigue and disrupts neuroimmune homeostasis. We further show that regular napping, even in an ecological, non-standardized context, can effectively limit these adverse effects by maintaining neurotrophic support (BDNF) and reducing pro-inflammatory signaling (IL-1β, IL-6). Taken together, our findings highlight the practical importance of napping. In an increasingly sleep-deprived society, it represents a simple yet powerful biological countermeasure to help preserve both long-term brain health and day-to-day cognitive performance.

## CRediT authorship contribution statement

**Sarah Belhout:** Writing – original draft, Formal analysis. **Laura Poudevigne:** Investigation, Formal analysis. **Lili Mouton:** Investigation, Formal analysis. **Aurélie Servonnet:** Supervision, Methodology. **Christine Le Coz:** Investigation. **Mariette Gaudin:** Investigation. **Mounir Chennaoui:** Writing – review & editing. **Damien Léger:** Writing – review & editing. **Arnaud Rabat:** Writing – review & editing, Writing – original draft, Validation, Supervision, Methodology, Conceptualization. **Brice Faraut:** Writing – review & editing, Writing – original draft, Validation, Supervision, Methodology, Investigation, Formal analysis, Conceptualization.

## Ethics declaration

Written informed consent to take part in the study and to publish the article has been obtained from all participants or their legal representatives. The privacy rights of participants have been observed.

This study was performed in compliance with relevant laws, regulatory frameworks and guidelines where the research took place. This study was approved by the Comité de protection des personnes Est III; CHRU Nancy, 54511 VANDOEUVRE LES NANCY, France. (Approval No. ID RCB: 2022-A00698-35)

## Declaration of competing interest

The authors declare that they have no known competing financial interests or personal relationships that could have appeared to influence the work reported in this paper.

## Data Availability

Data will be made available on request.
