# Supplementary material for: Regular napping mitigates the onset of cognitive fatigue and protects against stress–inflammatory responses to real-world sleep debt
Source: Brain Behav Immun Health. 2026 Oct 13;58:101390. doi: 10.1016/j.bbih.2026.101390 (PMC13696335; doi:10.1016/j.bbih.2026.101390)
Supplement: Supplementary file 1 [file mmc1.docx]

**Supplementary material 1:**

After providing written informed consent, participants underwent a brief medical history and physical examination to rule out neurological, psychiatric, or sleep disorders, substance or alcohol dependence, and clinically significant anxiety or depression (Hospital Anxiety and Depression score > 16). Additional exclusion criteria included an extreme morning chronotype; major medical or psychiatric conditions such as cardiovascular disease, neurodegenerative disorders, epilepsy, or substance misuse within the past two years (DSM-5 criteria); diagnosed sleep disorders or excessive daytime sleepiness (Epworth Sleepiness Scale > 16); severe depressive or mood symptoms (HAD > 16); treatments potentially affecting vigilance, high stimulant or tobacco use (caffeine > 500 mg/day or > 10 cigarettes/day); pregnancy; shift work, recent jet lag, or frequent trans meridian travel; use of medications likely to affect sleep; intensive physical activity (> 5 h/week); visual impairment or color blindness; difficulty completing cognitive tests; BMI > 29 kg/m²; or age > 40 years. Administrative exclusions comprised lack of national health-insurance affiliation, current medical leave, participation in another research protocol within the previous 30 days, receipt of research compensation exceeding the legal limit or failure to provide written informed consent.

**Supplementary material 2:**

The actigraph was set to sample movements in 1-min periods. Data were recorded via a sensor placed within the wrist device and data analysis (Actiwave MW8 software) assessed the different parameters of the activity/rest cycle. Sleep start and sleep-wake states were assessed independently and as follows: sleep-wake discrimination algorithm is based solely on the presence (or absence) of movement while sleep start is defined using the first immobile block of at least 10 min following bedtime containing no more than one epoch of movement. The sleep diary was used to corroborate the actigraphic data. The following parameters were extracted following routines implemented in the actigraphy software^76^: TST that is the sum of all sleep epochs; bedtime, get-up time, and sleep efficiency^77^. Daytime sleep periods were detected and retained only for the Sleep-Debt + Nap group.

**Supplementary material 3:**

Once thawed, saliva samples were centrifuged again prior to analysis. Salivary cortisol concentrations were measured by enzyme immunoassay (Salivary Cortisol Enzyme Immunoassay kit; Salimetrics®, State College, PA, USA). For interleukin-6 (IL-6) and IL-1 β, salivary concentrations were determined using an enzyme linked immunosorbent assay (Salivary Il-6 saliva kit and Salivary IL1- β kit; Salimetrics®). The intra- and inter-assay coefficients of variation were 3/3 % for cortisol, and IL-6, and 3–6 % for IL-1β. The analytical ranges were 0.33–83 nmol/L for cortisol, 0.35 -500 pg/mL for IL-6, and 3.13–200 pg/mL for IL-1β. All assays were performed in duplicate, and the mean values were used for subsequent statistical analyses. BDNF was assessed in saliva with the DRG BDNF ELISA kit (BDNF ELISA – ref: EIA 5968). The saliva samples were diluted at ½, and the ELISA assays were performed according to the manufacturer’s instructions. The detection range of the kit was 7.8-500pg/mL and intra-and inter-assay CVs were <5%.
